# Fall in Vitamin D Levels during Hospitalization in Children

**DOI:** 10.1155/2014/291856

**Published:** 2014-03-30

**Authors:** Devi Dayal, Suresh Kumar, Naresh Sachdeva, Rakesh Kumar, Meenu Singh, Sunit Singhi

**Affiliations:** ^1^Department of Pediatrics, Postgraduate Institute of Medical Education and Research, Chandigarh 160012, India; ^2^Department of Endocrinology, Postgraduate Institute of Medical Education and Research, Chandigarh 160012, India

## Abstract

Plasma levels of 25-hydroxyvitamin D [25(OH)D] were measured by competitive Electrochemiluminescence Immunoassay (ECLIA) in 92 children (67 boys, 25 girls) aged 3 months to 12 years at admission to hospital (timepoint 1, T1) and at discharge (timepoint 2, T2). There was a significant fall in the mean 25(OH)D from T1 (71.87 ± 27.25 nmol/L) to T2 (49.03 ± 22.25 nmol/L) (mean change = 22.84 nmol/L, *P* value = 0.0004). Proportion of patients having VDD (levels <50 nmol/L) at admission (25%, 23/92) increased significantly at the time of discharge (51.09%, 47/92) (*P* = 0.0004). There was a trend towards longer duration of hospital stay, requirement of ventilation and inotropes, development of healthcare-associated infection, and mortality in vitamin D deficient as compared to nondeficient patients though the difference was statistically insignificant. In conclusion, vitamin D levels fall significantly and should be monitored during hospital stay in children. Large clinical studies are needed to prospectively evaluate the effect of vitamin D supplementation in vitamin D deficient hospitalized children on various disease outcome parameters.

## 1. Introduction

Hospitalized children are prone to vitamin D deficiency (VDD) or exacerbate their existing deficiency due to multitude of reasons; many have VDD at the time of hospitalization due to widespread VDD, no additional vitamin D source due to poor oral intake and any sun exposure, and lack of practice of supplementation during hospitalization. Vitamin D has several skeletal as well as extraskeletal functions that include immunomodulation and cardioprotection as well as improvement of antimicrobial action, all of which may affect the outcomes in hospitalized patients [1, 2]. Several recent studies in adults suggest that VDD is associated with longer hospital stay and increases in morbidity and mortality [3–5]. A few intervention trials suggest some beneficial effect of vitamin D supplementation on outcomes in hospitalized vitamin D deficient patients although definite evidence is lacking [6, 7]. These studies have predominantly been conducted on patients admitted to intensive care units (ICUs). A few studies in pediatric population have shown similar results underlining the importance of estimating vitamin D levels in children with critical illness [8, 9]. All these studies have correlated the initial vitamin D levels with disease parameters. There is little information available on the change in vitamin D levels during hospitalization in children although one previous study done on adults indicates a significant fall in levels [10]. We thus planned to study the prevalence of VDD in children at the time of admission and the change in levels after a variable period of stay in a general pediatrics unit of our hospital.

## 2. Material and Methods

### 2.1. Subjects and Protocols

Children aged 3 months–12 years admitted to a General Pediatrics unit of a tertiary care hospital in North India were prospectively enrolled over a period of 4 months (July–October, 2012). Children with renal and liver disorders and malabsorption syndromes, those receiving multivitamins or calcium supplements and antiepileptic drugs, and those hospitalized for <7 days were excluded. The Ethics Committee of the Institute approved the study. Informed written consent from the parents/caretakers was obtained and assent of the child was taken wherever possible before recruitment into the study.

Demographic data, medical history, anthropometric measurements, and detailed physical examination were recorded at admission. Clinical signs suggestive of VDD if present and risk factors for VDD were also noted. Blood samples were taken for analyses of 25-hydroxyvitamin D [25(OH)D], calcium, phosphorus, and alkaline phosphatase at admission (timepoint 1, T1) and at discharge from hospital (timepoint 2, T2). In patients who died, their most recent stored sample was used to assess 25(OH)D and other biochemical parameters. Patients were followed up till discharge from hospital or death.

### 2.2. Laboratory Methods

Blood samples for 25(OH)D measurement were collected in heparinized amber colored glass vials to prevent its photodegradation. Plasma was extracted after centrifugation and stored at −20°C until analyzed. Plasma total 25-OHD measurement was done by competitive Electrochemiluminescence Immunoassay (ECLIA) (E-2010, Roche Diagnostics, Germany) using kits, calibrators, and controls from the same manufacturer. The detection limit of the method used is 3.75 nmol/L (1.5 ng/mL).

### 2.3. Definition of Vitamin D Deficiency and Insufficiency

Depending on their 25(OH)D level, patients were classified into 3 categories [11, 12]:

1. vitamin D deficiency: 25(OH)D level <50 nmol/L (<20 ng/mL);
2. vitamin D insufficiency: 25(OH)D levels 50–75 nmol/L (20–30 ng/mL);
3. vitamin D sufficiency: 25(OH)D levels >75 nmol/L (>30 ng/mL).

### 2.4. Outcomes

Primary outcome was the prevalence of VDD at timepoints 1 and 2 and secondary outcomes were relationship of 25(OH)D with clinically important outcomes including duration of stay in hospital, requirement of ventilation (invasive or noninvasive methods to assist or replace spontaneous breathing, e.g., continuous positive airway pressure, manual ventilation, and mechanical ventilation), requirement of inotropes (agents used to augment cardiac contractility, e.g., dopamine, dobutamine, adrenaline, nor-adrenaline, and milrinone), incidence of nosocomial infection (any infection that a patient acquired within the hospital setting that was not present at the time of admission), and death.

## 3. Data Analysis

Appropriate data entry and statistical analysis were performed on Microsoft Excel 2007 (Microsoft, Redmond, WA) and SPSS software version 15 (SPSS, Inc, Chicago, IL). Demographic variables were tested with chi-square test and reported as mean, SD, range, and percentages, as applicable. Dichotomous outcomes were compared by chi-square test or Fisher's exact test, as applicable. Continuous variables were compared by Student's *t*-test. Association of vitamin D level with patient characteristics and outcome variables was measured by using chi-square test and Fisher's tests for categorical variables and *t*-tests, Wilcoxon rank sum, Mann-Whitney, or Kruskal-Wallis tests for continuous variables, where appropriate. The regression of T1 and T2 on hospital duration was also calculated. All tests were two-tailed and *P* < 0.05 was taken as significant.

## 4. Results

### 4.1. Clinical Characteristics of Study Population

Over the study period, 146 patients were admitted to the unit; out of these 92 patients were enrolled. Reasons for nonenrollment included refusal of consent (*n* = 8), age <3 months (*n* = 9), renal disorder (*n* = 6), liver disorder (*n* = 4), malabsorption syndrome (*n* = 3), on calcium and vitamin D supplements (*n* = 4), on antiepileptic drugs (*n* = 3), and duration of stay <7 days (*n* = 17). Their clinicodemographic characteristics and final outcomes are shown in Table 1.

### 4.2. Prevalence of Vitamin D Deficiency and Insufficiency

There were a significant increase from T1 to T2 in the percentage of 25(OH)D deficient patients (25% versus 51.09%, *P* = 0.0004) and a decrease in 25(OH)D sufficient patients (30.43% versus 15.22%, *P* = 0.02) (Table 2). Similarly the fall in the mean levels of 25(OH)D from T1 to T2 (71.87 ± 27.25 versus 49.03 ± 22.25 nmol/L, mean change = 22.84 nmol/L, *P* value = 0.0004) was statistically significant (Table 3).

### 4.3. Distribution of Serum 25(OH)D, Calcium, Phosphorus, and Alkaline Phosphatase

Total calcium and phosphorus levels were also significantly lower at T2, whereas alkaline phosphatase levels were similar at the 2 timepoints (Table 3).

The mean levels of 25(OH)D at timepoint 1 and timepoint 2 were lower in cases who required ventilation, received inotropes, developed nosocomial sepsis, and those who died though the difference was not statistically different (Table 4). Similarly when the comorbidities were pooled, the levels of 25(OH)D at the 2 timepoints were lower in patients with either of these comorbidities as compared to those without any comorbidity but the differences were statistically insignificant (66.37 ± 24.67 nmol/L versus 72.90 ± 28.95 nmol/L, *P* = 0.71 and 47.55 ± 22.95 nmol/L versus 49.52 ± 22.27 nmol/L, *P* = 0.48, resp.).

Vitamin D deficiency at timepoint 1 was more likely to be associated with failure to thrive (*P* = 0.03), short stature (*P* = 0.025), exclusive breastfeeding (*P* = 0.03), inadequate sun exposure (*P* = 0.02), calcium levels (*P* = 0.009), and alkaline phosphatase levels (*P* = 0.008) (Table 5). However, multivariate analysis did not show an independent association of any of these factors with VDD at timepoint 1. On linear regression, 25(OH)D levels at timepoints 1 and 2 were inversely correlated with duration of hospital stay (in days) though statistically insignificant (*r* ^2^ = 0.011, *P* = 0.31 and *r* ^2^ = 0.012, *P* = 0.30, resp.). Vitamin D deficient patients were supplemented with adequate doses of vitamin D and calcium at the time of discharge. Of the 6 patients who died during the study period, the underlying diagnoses were tubercular meningitis in 2, acute viral meningoencephalitis in 1, diabetic ketoacidosis with disseminated fungal infection in 1, disseminated staphylococcal disease in 1 and pneumonia with nosocomial infection in 1.

## 5. Discussion

The data on prevalence of VDD obtained in this study is similar to the data in control subjects of a previously conducted study by us on vitamin D levels in type 1 diabetes patients [13]. The present study demonstrated a significant change in vitamin D status during hospitalization. The mean 25(OH)D levels fell by almost one-third and the proportion of vitamin D deficient patients doubled over a hospital duration of approximately 2 weeks. This fall in 25(OH)D levels may be related to factors that operate in a hospitalized child. Vitamin D deficiency is common in India as well as around the world and many children may be vitamin D deficient at the time of hospitalization [14, 15]. Poor oral intake and intestinal absorption due to illness and no sun exposure may exacerbate the existing deficiency. Additionally there is lack of practice of supplementation during hospitalization. The exact reasons for the significant fall in 25(OH)D concentrations (half-life approximately 3 weeks) within days of hospitalization are unclear. Recent studies have indicated that the catabolic pathways of vitamin D become predominant during critical illness and infection [9]. Also lower levels of vitamin D binding proteins due to interstitial extravasation resulting from increased vascular permeability during inflammatory responses and decreased synthesis may contribute to lower 25(OH)D levels [16]. In addition the dilutional effect of fluid supplementation during hospitalization may reflect in lower 25(OH)D concentrations. While the prevalence of VDD in hospitalized individuals is well documented [3–5, 8, 9, 17, 18], the clinical significance of this deficiency remains to be fully ascertained. Initial studies indicated that VDD affects most outcome parameters in adults as well as children admitted to ICUs and suggested supplementation to reduce morbidity and mortality [3–5, 8, 9, 17, 18]. Other studies, however, have failed to produce similar data on outcomes [19, 20]. We also could not demonstrate significant change attributable to vitamin D status on clinically relevant outcome parameters in our patients. Similarly the intervention trials with vitamin D have shown mixed results with most showing insignificant effects on various hospital outcomes [6, 7]. It is opined that this lack of significant effect on outcomes may be related to caveats in study design, vitamin D doses, and administration routes [6, 7, 21]. An alternative explanation may be related to efficiency of the vitamin D induced local as well as systemic immunity [22, 23]. A robust immune response is expected in those who are vitamin D replete at the time of acquiring infection and may help eliminate infection by augmenting the antibiotics' ability. The process of attaining this vitamin D sufficient state (with normal elicitation of vitamin D related immunity) after supplementation may take several days depending on vitamin D pharmacokinetics in body [24]. Thus, the initial supplementation in hospitalized persons may not have significant effect on the outcomes of presenting illness but may be beneficial in preventing the hospital-related morbidity if hospitalization becomes prolonged. In this context the fall in 25(OH)D levels shown in our study may be proposed as a ground for vitamin D supplementation in all hospitalized children to maintain an optimal 25(OH)D status. It will also be interesting to see the impact of routine childhood vitamin D supplementation aimed at achieving an optimal vitamin D status on hospitalization rates and hospital outcomes in children in future.

The decrease in calcium and phosphorus levels in our study paralleled the decrease in 25(OH)D levels as expected [25]. A higher percentage of vitamin D deficient children had history of exclusive breast feeding and inadequate sun exposure, risk factors observed in previous studies as well [14]. The clinical signs of VDD were seen in far less number of cases than biochemical VDD as they develop only when the VDD persists for some duration [26]. So, relying only on clinical signs will miss a significant number of cases with VDD, which would otherwise be identified by measuring 25(OH)D levels. Our data collected during summer and monsoon seasons may have underestimated the actual prevalence of VDD in the studied subjects as 25(OH)D levels fluctuate throughout the year [27].

The data on change in vitamin D status during hospitalization is virtually nonexistent. Only one previous study conducted prospectively on adult patients demonstrated significant decrease in 25(OH)D levels in all patients after 3 days that remained significantly lower through 10 days in the ICU [10]. Our study appears to be the first one to have documented a fall in 25(OH)D levels in hospitalized children.

In conclusion, there are a significant fall in vitamin D levels over short duration hospitalization in children and a need to routinely screen all hospitalized children for VDD. Large multicentered trials are needed to prospectively evaluate the effect of vitamin D supplementation in vitamin D deficient hospitalized children on various disease outcome parameters.

## Figures and Tables

**Table 1:** Clinicodemographic characteristics and final outcomes of patients.

Characteristics	Total patients, *n* = 92
Mean age (years) (range, ±SD)	5.27 (0.3–12, 4.32)
Sex (male : female)	67 : 25
Weight (kgs), mean (range, ±SD)	15.45 (3–48, 9.9)
Height (cms), mean, (range, ±SD)	99.55 (52–156, 30.57)
Failure to thrive, *n* (%)	12 (13.04)
Short stature, *n* (%)	9 (9.78)
Diagnosis	
CNS infections, *n* (%)	16 (17.39)
Sepsis, *n* (%)	15 (16.3)
Pneumonia, *n* (%)	14 (15.22)
Cardiac disease, *n* (%)	13 (14.13)
Diabetes mellitus/diabetic ketoacidosis	9 (9.78)
Malaria, *n* (%)	5 (5.43)
DSS, *n* (%)	3 (3.26)
Other, *n* (%)	17 (18.48)
Signs of rickets	8 (8.69)
Rachitic rosary, *n* (%)	6 (6.52)
Frontal bossing, *n* (%)	6 (6.52)
Harrison sulcus, *n* (%)	5 (5.43)
Wrist widening, *n* (%)	5 (5.43)
Wide anterior fontanelle, *n* (%)	4 (4.35)
Double malleolus, *n* (%)	3 (3.26)
Craniotabes, *n* (%)	2 (2.17)
Bowing of legs, *n* (%)	2 (2.17)
Risk factors for vitamin D deficiency	
Family size, mean (range, ±2SD)	4.4 (3–8, 1.1)
Birth order, mean (range, ±2SD)	2.02 (1–6, 1.0)
Exclusive breast feeding, *n* (%)	51 (55.43)
Sun exposure, *n* (%)	47 (51.1)
Dark skin color, *n* (%)	7 (7.61)
Final outcomes	
Duration of stay in hospital (in days), mean (range, ±SD)	13.87 (8–23, 3.52)
Required ventilation, *n* (%)	21 (22.83)
Required inotropes, *n* (%)	15 (16.30)
Nosocomial sepsis, *n* (%)	9 (9.78)
Died, *n* (%)	6 (6.52)

**Table 2:** Distribution of patients in four categories according to 25(OH)D levels at timepoint 1 and timepoint 2.

25(OH)D levels (nmol/L)	Timepoint 1	Timepoint 2	*P* value
<50, *n* (%)	23 (25)	47 (51.09)	0.0004
50–75, *n* (%)	41 (44.56)	31 (33.69)	0.173
<75, *n* (%)	64 (69.57)	78 (84.78)	0.02
>75, *n* (%)	28 (30.43)	14 (15.22)	0.02

**Table 3:** Levels of 25(OH)D, calcium, phosphate, and alkaline phosphatase in study population at timepoint 1 and timepoint 2.

Characteristics	Timepoint 1	Timepoint 2	*P* value
25(OH)D level (nmol/L), mean (range, ±SD)	71.87 (20–167, 27.25)	49.03 (7.5–110, 22.25)	0.000
Calcium (mg/dL), mean (range, ±SD)	8.79 (5.9–12, 0.86)	8.53 (5–12, 0.93)	0.02
Phosphorus (mg/dL), mean (range, ±SD)	4.60 (2–6, 1.07)	4.29 (2–5, 1.17)	0.002
Alkaline phosphatase (IU/L), mean (range, ±SD)	229 (71–1004, 160)	236 (80–1135, 153)	0.32

**Table 4:** Mean (±SD) 25(OH)D levels at timepoints 1 and 2 among patients who required ventilation and inotropes, those who developed nosocomial sepsis, and those who died versus those who did not.

Characteristics	Yes	No	*P* value
Required ventilation, *n* (%)	21 (22.83)	71 (77.17)	
25(OH)D levels at timepoint 1	65.7 (21.1)	76.55 (28.95)	0.19
25(OH)D levels at timepoint 2	45.86 (20.15)	52.15 (23)	0.42
Required inotropes, *n* (%)	15 (16.30)	77 (83.70)	
25(OH)D levels at timepoint 1	66.88 (27.15)	75.91 (27.12)	0.16
25(OH)D levels at timepoint 2	44.62 (24.75)	53.78 (21.82)	0.32
Nosocomial sepsis, *n* (%)	9 (9.78)	83 (90.22)	
25(OH)D levels at timepoint 1	64.67 (27)	78.9 (29.4)	0.22
25(OH)D levels at timepoint 2	47 (27.8)	51 (28.3)	0.13
Died, *n* (%)	6 (6.52)	86 (93.48)	
25(OH)D levels at timepoint 1	65.8 (15.47)	75 (27.7)	0.16
25(OH)D levels at timepoint 2	42.85 (19.47)	56.15 (22.4)	0.17

**Table 5:** Comparison of various variables between vitamin D deficient and nondeficient children at timepoint 1.

Characteristics	Deficient, *n* = 23	Non-deficient, *n* = 69	*P* value
Mean age (±SD) (years)	5.1 (4.4)	5.36 (4.32)	0.73
Boys, *n* (%)	14 (60.87)	53 (76.81)	0.13
Girls, (%)	9 (39.13)	16 (23.19)	
Weight (in kgs), mean (±SD)	15.3 (10.91)	15.59 (9.64)	0.93
Height (in cms), mean (±SD)	97.43 (30.83)	100.2 (30.67)	0.70
Failure to thrive, *n* (%)	6 (26.09)	6 (8.69)	0.03
Short stature, *n* (%)	5 (21.74)	4 (5.80)	0.02
Risk factors for vitamin D deficiency			
Family size, mean (range, ±SD)	4.30 (1.03)	4.42 (1.15)	0.92
Birth order, mean (range, ±SD)	1.87 (0.96)	2.07 (1.02)	0.41
Exclusive breast feeding, *n* (%)	17 (73.91)	34 (49.27)	0.03
Adequate sun exposure, *n* (%)	7 (30.43)	40 (57.97)	0.02
Dark skin color, *n* (%)	4 (17.39)	3 (4.34)	0.25
Biochemical parameters			
Vitamin D levels (nmol/L), mean (±SD)	48.45 (22)	89.92 (24.1)	0.000
Calcium (mg/dL), mean (±SD)	8.39 (0.95)	8.93 (0.79)	0.009
Phosphorus (mg/dL), mean (±SD)	4.46 (1.0)	4.74 (0.92)	0.66
Alkaline phosphatase (units/L), mean (±SD)	309 (221)	149 (141)	0.008
Outcomes			
Hospital stay (in days), mean (±SD)	14.70 (3.21)	13.12 (3.59)	0.74
Required ventilation, *n* (%)	8 (34.78)	13 (18.84)	0.11
Required inotropes, *n* (%)	5 (21.74)	10 (14.49)	0.41
Nosocomial sepsis, *n* (%)	4 (17.39)	5 (7.24)	0.15
Died, *n* (%)	3 (13.04)	3 (4.35)	0.14
